# Comparison of survival between unilocular cystic and purely solid renal cell carcinoma

**DOI:** 10.1038/s41598-022-16856-2

**Published:** 2022-07-27

**Authors:** Yapeng Wang, Xiaoyu Niu, Lihui Wang, Yunlong Li, Baoping Qiao

**Affiliations:** Department of Urology, The First Affiliated Hospital of Zhengzhou University, Zhengzhou, 450052 China

**Keywords:** Cysts, Urological cancer, Renal cancer

## Abstract

To evaluate clinicopathological features and survival outcomes of unilocular cystic renal cell carcinoma (ucRCC) compared with purely solid renal cell carcinoma (sRCC), and to evaluate the oncologic aggressiveness of ucRCC. The relevant data of 957 patients with sporadic unilateral renal cell carcinoma (RCC) underwent surgical treatment in 2 institutions from Jan 2014 to Oct 2018 were obtained. We excluded multilocular cystic renal neoplasm of low malignant potential (MCRNLMP), RCC with multilocular cysts and necrotic RCC. 74 ucRCCs were identified by pathology reports. We performed propensity score matching (PSM) and eventually selected 144 sRCCs. The clinicopathological features and survival outcomes were compared properly. After PSM, age, BMI, Charlson Comorbidity Index, and postoperative Chronic Kidney Disease grade were not significantly different. Both overall survival and progression-free survival of ucRCC were significantly better than sRCC by the log-rank test. Twenty-five cases of sRCCs were in the pT3 or pT4 stage, while no pT3 or pT4 tumors were found in ucRCCs. Fuhrman grade and lymphatic metastasis were found to be significant prognostic factors for the overall survival of ucRCC. Unilocular cystic RCC has a lower Fuhrman grade and pathological stage and a better prognosis compared with solid RCC. Patients with ucRCC still probably have lymphatic metastasis at surgery and may have postoperative metastasis, which is different from MCRNLMP. We recommend that the diagnosis of ucRCC should be reflected in pathology report. Different subtype of cystic RCC should be taken into consideration in counseling and management.

## Introduction

Cystic renal cell carcinoma (cRCC) was first defined as renal carcinoma with cystic or cystic-solid changes on imaging and confirmed by pathology and classified into 4 subtype by Hartman et al.^1^.

In 2016, the World Health Organization redefined multilocular cystic renal cell carcinoma, a subtype of cRCC, as the multilocular cystic renal neoplasm of low malignant potential (MCRNLMP) based on the excellent prognosis wherein more than 200 patients were followed up for more than 5 years without recurrence or metastasis^2–4^. Some studies have also found that most of MCRNLMP have Von Hippel-Lindau (VHL) mutations and 3p deletions, which suggests that they are related to clear cell renal cell carcinoma at the molecular level^2,3,5,6^. Compared with solid renal cell carcinoma (sRCC), the MCRNLMP has a very distinctive histology and an excellent prognosis. In terms of tumor morphology, we can classify renal cell carcinoma (RCC) into the following types: MCRNLMP; RCC with a multilocular cystic component (not meeting the diagnosis of MCRNLMP); unilocular cRCC and purely solid RCC.

MCRNLMP is a morphology and prognosis distinctive subtype of cRCC and accounts for only about 1% of RCC, while the whole cRCC accounts for 15%^2,4,5^. Many previous studies failed to identify it separately, but classified MCRNLMP together with all other types as the cRCC, and investigated their outcomes^6–10^.

Although those studies have discovered that the cystic morphology of RCC is associated with a good prognosis^6–10^, other types of cRCC actually do not have as good prognosis as MCRNLMP^2,11,12^. In that way, those previous studies have made incorrect judgments on the prognosis of many other types of cRCC besides MCRNLMP, especially unilocular cystic renal cell carcinoma (ucRCC). Few studies have previously classified this type of tumors, which is completely different from MCRNLMP, into a separate category for survival research.

In this review, we intend to analyze the clinicopathological data of ucRCC and compare it with those of purely solid RCC to improve clinician’s understanding of this subtype of cRCC and provide new perspective for clinical management decisions and follow-up.

## Materials and methods

### Inclusion and exclusion criteria

**Figure 1:**
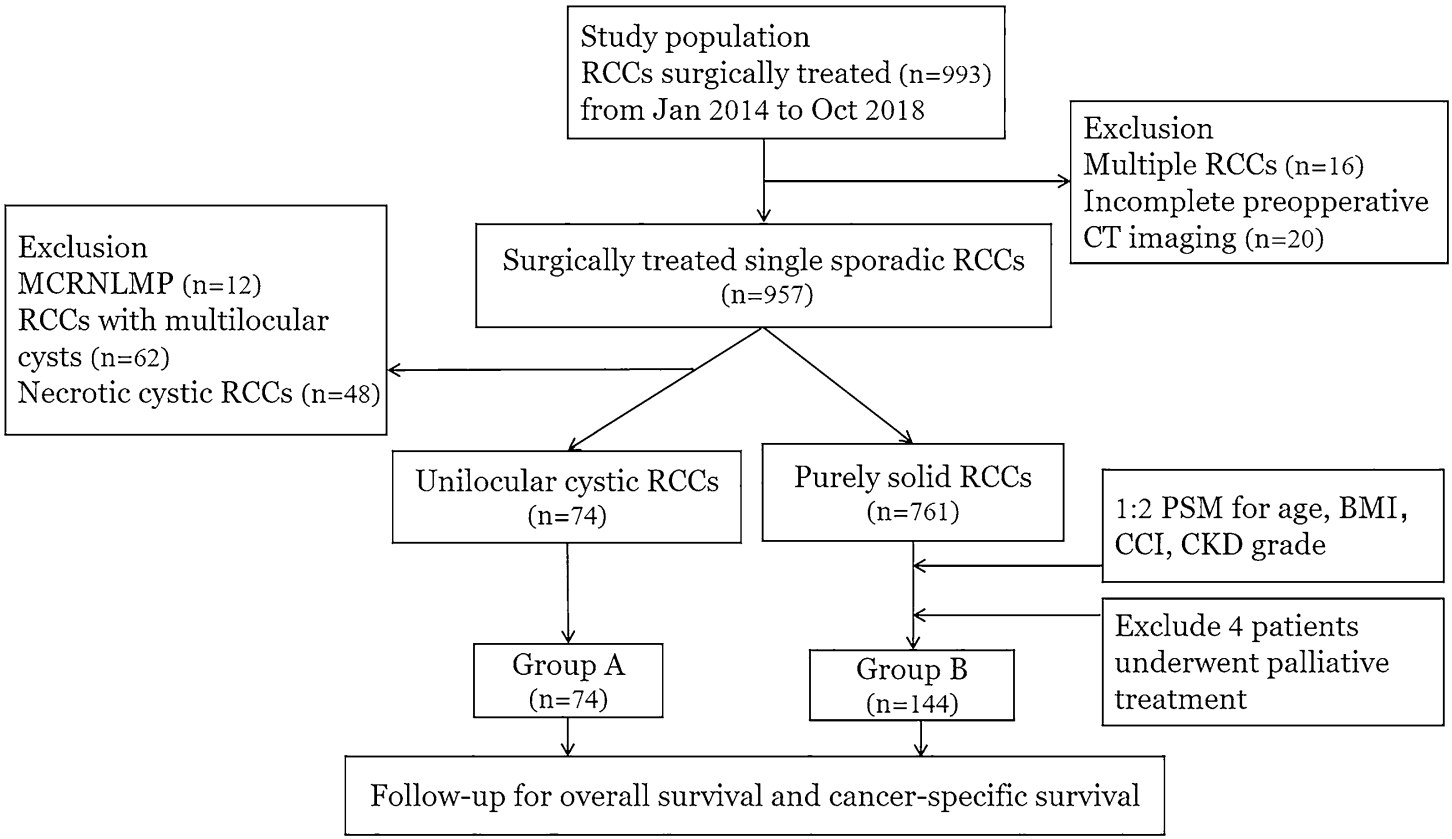
Flowchart of enrolling procedure.

The date of 993 patients was obtained from January 2014 to October 2018 at two different institutions with approval from Ethics Committee of Zhengzhou University. We excluded 16 cases of multifocal RCC (including 6 cases of VHL syndrome), 20 cases with incomplete preoperative images, 12 cases of MCRNLMP, 62 cases of RCC with multilocular cystic component (not meeting the diagnosis of MCRNLMP), 48 cases with necrotic cysts confirmed by surgical pathology (including 9 cases misdiagnosed as true cysts by radiologists). Finally, 835 patients were included in this study, among which 74 cases were identified as unilocular non-necrotic cRCC by surgical pathology reports and placed in group A (Fig. 1). The optimistic cut off value of the cystic component for determining cRCC is different in various studies (ranging from 5 to 75%)^6,8,13^, and the number of deaths in RCC with cystic components was relatively small in our study. Therefore, we were not able to select a reliable cut off for unilocular cRCC but included all RCC with obvious single cysts on preoperative CT imaging and confirmed by pathology reports as non-necrotic in group A. Meanwhile, we used Stata ® to perform 1:2 propensity score matching (PSM; Match tolerance set at 0.05, Maximize execution performance) by age, BMI, Charlson Comorbidity Index (CCI), and postoperative Chronic Kidney Disease (CKD) grade (which was evaluated from the estimated glomerular filtration rate at the 6th month) using the abbreviated Modification of Diet Renal Disease Study Group in 761 cases of pathologic confirmed solid RCC, and randomly identified 148 patients. After excluding 4 patients who had distant metastases at surgery and underwent cytoreductive surgery, 144 cases were included in group B for comparison.

### Image measurements

All renal lesions underwent CT scan (Philips Diamond Select Brilliance 64-Slice CT scanner) and imaging data was reviewed by experienced radiologists unaware of the clinical data. The record content included: R.E.N.A.L. score and Bosniak classification of the lesions, the respective volume of the entire tumor and cystic components calculated by computer software multiplying the area of all sections by the thickness of each layer, and tumor diameter that was the average of the diameter on each axis of the tumor. Although our study relied on pathology results to diagnose unilocular cRCC, we also required radiologists to judge the nature of cysts when reviewing CT. The criteria radiologists applied to determine the true cystic component were: 1) The CT-value in the capsule being less than 20 Hu and unmixed density at routine scan. 2) There was no obvious enhancement in the capsule during the cortical phase (≤ 40Hu).

### Statistics

All statistical analyses were conducted with SPSS®, version 26.0. Continuous variables were presented as mean ± SD and compared by the t test. Categorical variables were described by frequencies with proportions and compared by chi-square and Wilcoxon rank sum test as appropriate. The primary end points were overall survival (OS) and progression-free survival (PFS). The median follow-up time is 4.57 years (1.25–6.74). The overall survival (OS) and progression-free (PFS) were estimated by the Kaplan–Meier curves, and differences between curves were assessed with log rank test. Moreover, cox proportional hazards models of univariate and multivariate analyses were applied to identify factors associated with overall survival for ucRCC, expressed using hazard ratio (HR), 95% confidence interval (95% CI). The factors with *p* value less than 0.1 in univariate regression analysis were included in multivariate regression analysis, and all other results were considered statistically significant only with *p* < 0.05.

### Ethics approval and informed consent

All methods in this study involving human participants were performed in accordance with the ethical standards of the institutional research committee and were permitted by Ethics Committee of Zhengzhou University, with no living animals involved. This study is a retrospective study, Ethics Committee of Zhengzhou University therefore waived the informed consent.

### Human and/or animals participants

This study is a review of existing data and does not involve any studies with human participants or animals performed by other authors.

## Results

### Clinicopathological features and radiology characteristics

**Figure 2:**
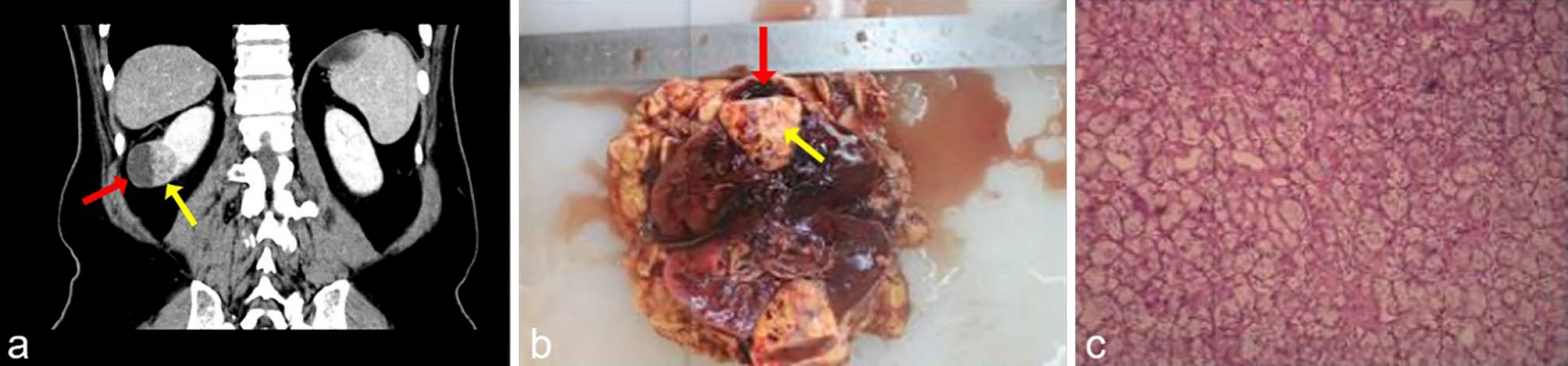
A 60-year-old male with a unilocular cRCC. (**a**) Postcontrast CT: unenhanced cyst fluid (red arrow) and solid component (yellow arrow). (**b**,**c**) Pathology report: Non-necrotic cyst and Fuhrman grade II clear cell carcinoma of the solid component.

**Figure 3:**
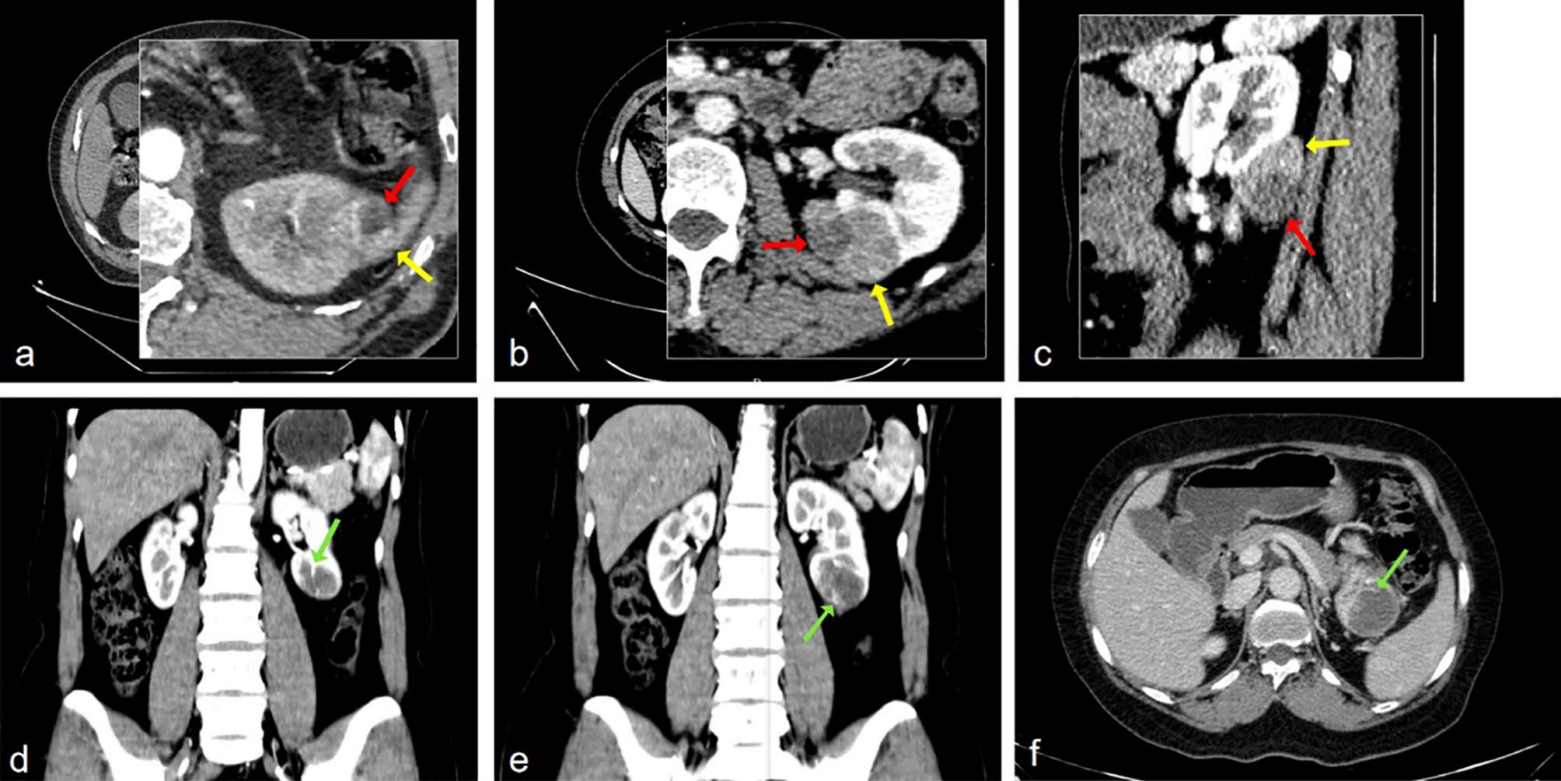
(**a**,**b**,**c**) Postcontrast CT: cystic (red arrow) and solid (yellow arrow) components of unilocular cystic clear cell RCC. (**e**,**f**,**g**) irregular internal structures (green arrow) in the unilocular cystic papillary RCC.

Tables 1 and 2 summarize and compare the clinicopathological and radiological data of purely solid RCC and unilocular cRCC. Table 3 shows characteristics of entire 761 sRCC patients. After PSM, there were no significant differences in age, BMI, CCI, and postoperative CKD grade between 2 groups. Gender composition, tumor diameter, R.E.N.A.L. score and surgical approach were not significantly different as well. The average diameter and volume of tumors in group A were 4.45 ± 1.10 cm and 100.63 ± 74.02 cm^3^ respectively, while those in group B were 4.55 ± 0.96 cm and 105.88 ± 67.20 cm^3^ respectively, and there with no significant difference (*p* = 0.521, *p* = 0.599). In terms of Fuhrman grade, only 2 cases in group A were G3, and there were no G4 tumors. However, G3 and G4 tumors in group B accounted for 36.2%, and the difference between 2 groups was significant (*p* < 0.001). The decision to perform lymphadenectomy for all patients was uniformly based on preoperative CT findings of enlarged lymph nodes. In group A, 5 patients underwent lymphadenectomy and 3 of them were pathologically confirmed to have lymphatic metastasis. In group B, 16 patients underwent lymphadenectomy, of which 9 had confirmed lymphatic metastasis. Sixty-six (89.2%) cases of group A were in the pT1 stage and the remaining 8 (10.8%) were in the pT2 stage. While no pT3 or pT4 tumors were found in group A. Meanwhile, 21 (14.6%) cases in group B were in the pT3 or pT4 stage. The difference between the 2 groups is statistically significant (*p* = 0.006). The most common histology type in the 2 groups was clear cell carcinoma (Fig. 2), but there were significantly more (*p* = 0.021) cases of papillary carcinoma in group A (12.2%) than in group B (2.8%). And a higher proportion of type I papillary RCC in group A compared to matched and total sRCC group was identified, though not statistically significant (*p* = 0.217). None of the patients in the 2 groups who underwent partial nephrectomy showed positive margins in the pathology reports. We also noticed that 26 (35.1%) unilocular cysts in group A had irregularly thickened inner walls greater than 2 mm and 11 (14.9%) cysts had irregular internal structures (Fig. 3). These structures only protrude into the cavity, but do not form a septum separating the cysts, which is different from the septa of multilocular cRCC. The average cystic proportion of tumors in group A was 33.08%, and the volume of smallest cyst only accounted for 4%, while the largest accounted for 78%.

**Table 1:** Demographic data of unilocular cystic and solid RCC.

	Unilocular cRCC *n* = 74	Solid RCC *n* = 144	*P* value
Age	52.04 ± 10.68	51.91 ± 10.97	*P* = 0.933
**Gender**			*P* = 0.400
Male	52 (70.3%)	93 (64.6%)	
Female	22 (29.7%)	51 (35.4%)	
**Location**			*P* = 0.577
Left	42 (56.8%)	76 (52.8%)	
Right	32 (43.2%)	68 (47.2%)	
BMI	25.34 ± 2.95	25.03 ± 2.94	*P* = 0.742
**Surgery approach**			*P* = 0.411
Partial nephrectomy	32 (43.2%)	54 (37.5%)	
Radical nephrectomy	42 (56.8%)	90 (62.5%)	
**CCI**			*P* = 0.568
< 5	55 (74.3%)	112 (77.8%)	
≥ 5	19 (25.7%)	32 (22.2%)	
**CKD**			*P* = 0.516
1 ~ 2	45 (60.8%)	94 (65.3%)	
3 ~ 4	29 (39.2%)	50 (34.7%)

**Table 2:** Pathology and radiology characteristics of unilocular cystic and solid RCC.

	Unilocular cRCC	Solid RCC	*P* value
	*n* = 74	*n* = 144	
**Fuhrman grade**			*P* < 0.001
G1	33 (44.6%)	8 (5.6%)	
G2	39 (52.7%)	84 (58.3%)	
G3	2 (2.7%)	45 (31.3%)	
G4	/	7 (4.9%)	
**Metastasis**			*P* = 0.501
N0M0	71 (95.9%)	135 (93.8%)	
N + and M0	3 (4.1%)	9 (6.2%)	
**pT**			*P* = 0.006
1	66 (89.2%)	113 (78.5%)	
2	8 (10.8%)	10 (6.9%)	
3	/	20 (13.9%)	
4	/	1 (0.7%)	
**Histology**			*P* = 0.021
Clear cell	64 (86.4%)	137 (95.1%)	
Papillary	9 (12.2%)	4 (2.8%)	
Chromophobe	1 (1.4%)	3 (2.1%)	
**Papillary subtype**			*P* = 0.217
Type I	7 (78%)	1 (25%)	
Type II	2 (22%)	3 (75%)	
Diameter of tumor mean ± SD, cm	4.45 ± 1.10	4.55 ± 0.96	*P* = 0.521
Volume mean ± SD, cm3	100.63 ± 74.02	105.88 ± 67.20	*P* = 0.599
**R.E.N.A.L. score**			*P* = 0.955
< 7	3 (4.1%)	5 (3.5%)	
7 ~ 9	25 (33.8%)	51 (35.4%)	
10 ~ 12	46 (62.2%)	88 (61.1%)	
**Bosniak classification**			
I	/	/	
II	/	/	
II F	2 (2.7%)	/	
III	26 (35.1%)	/	
IV	46 (62.2%)	/

**Table 3:** Characteristics of 761 patients with solid renal cell carcinoma.

	Solid RCC	Characteristic	Solid RCC
	n = 761		n = 761
Age	61.54 ± 9.32	**Metastasis**	
		N0M0	618 (51.2%)
**Gender**		N + and M0	68 (8.9%)
Male	480 (63.1%)	≥ M1	75 (9.9%)
Female	281 (36.9%)	**pT**	
**Location**		1	567 (74.5%)
Left	413 (54.3%)	2	49 (6.4%)
Right	348 (45.7%)	3	121 (15.9%)
BMI	26.24 ± 2.32	4	24 (3.2%)
**Surgery approach**		**Histology**	
Partial nephrectomy	268 (35.2%)	Clear cell	659 (86.6%)
Radical nephrectomy	493 (64.8%)	Papillary	29 (3.8%)
**CCI**		Chromophobe	32 (4.2%)
< 5	587 (77.2%)	Others	41 (5.4%)
≥ 5	174 (22.8%)	**Papillary subtype**	
**CKD**		Type I	12 (41.4%)
1 ~ 2	504 (66.3%)	Type II	17 (58.6%)
3 ~ 4	257 (33.8%)	Diameter of tumor	4.64 ± 0.68
**Fuhrman grade**		mean ± SD, cm	
G1	33 (4.3%)	**R.E.N.A.L.score**	
G2	419 (55.1%)	< 7	24 (3.2%)
G3	247 (32.5%)	7 ~ 9	242 (31.8%)
G4	62 (8.1%)	10 ~ 12	495 (65.0%)

### Survival analysis

**Figure 4:**
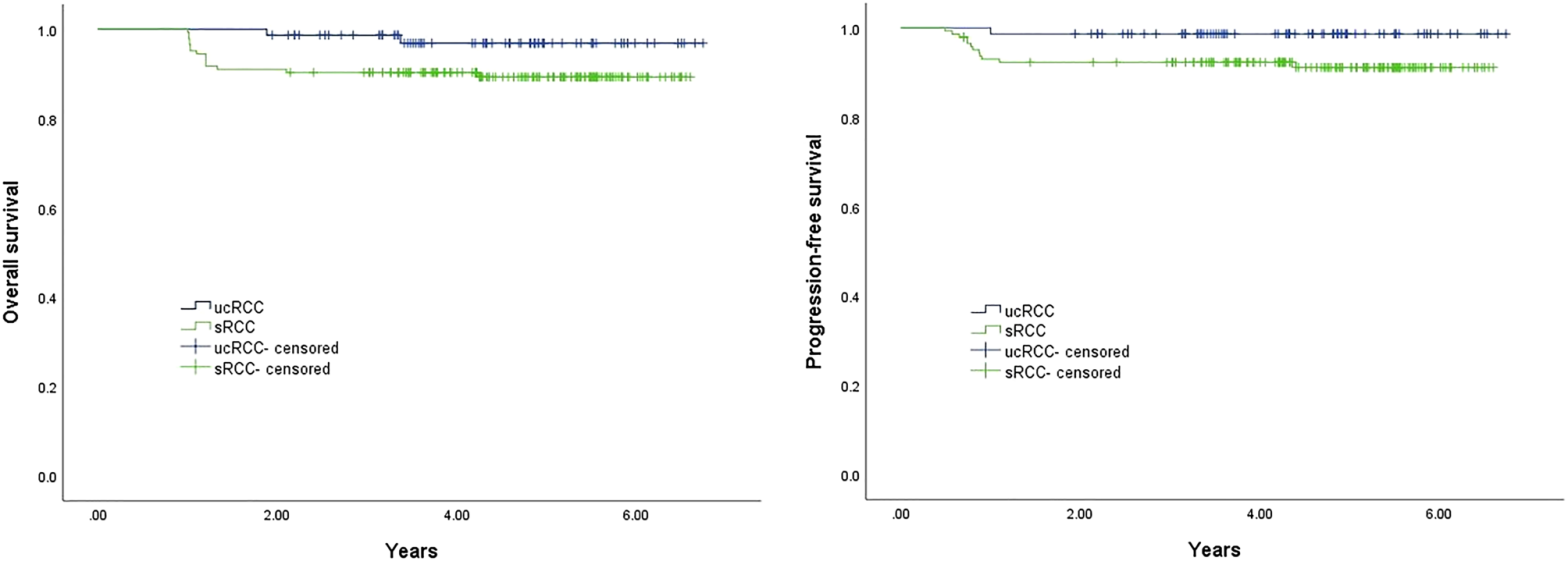
Kaplan–Meier curves of overall and progression-free survival for unilocular cystic and solid RCC.

Kaplan–Meier curves of the 2 groups as shown in Fig. 4. Both overall survival (*p* = 0.047) and progression-free survival (*p* = 0.040) of the unilocular cRCC were significantly better than the purely solid RCC by the log-rank test. Fourteen patients in group B died and 12 patients experienced recurrence or metastasis. A patient of group B was reported to have local recurrence 2 years post-partial nephrectomy and was treated with radical nephrectomy. Lumbar metastasis was found 2 years later and currently VEGFR inhibitors treatment is being administered. In group A, only 1 patient died of lung metastasis after radical nephrectomy. Meanwhile, of the 12 MCRNLMP patients in our original cohort, except one patient lost to follow-up, the remaining 11 patients are alive and have no recurrence or metastasis according to our recommended annual physical examination. Furthermore, we applied the Cox proportional hazards regression model to perform univariate and multivariate regression analysis of survival data (Table 4). The factors lymphatic metastasis and Fuhrman grade were identified to be significant predictors in univariate regression analysis of overall survival, though they were not statistically significant in the multivariate regression analysis.

**Table 4:** Cox regression analysis of overall survival for 74 patients.

	Univariate		Multivariate	
	HR (95% CI)	*P* Value	HR (95% CI)	*P* Value
Age	1.10 (0.92–1.30)	0.299	/	
CCI	2.81 (0.89–8.93)	0.079	5.62 (1.89–361.82)	0.149
CKD grade	62.77 (0.01–684.28)	0.383	/	
BMI	0.84 (0.48–1.46)	0.529	/	
Fuhrman grade	26.13 (1.89–361.82)	0.015	26.87 (0.76–952.54)	0.071
pT stage	9.08 (0.57–145.68)	0.119	/	
Lymphatic metastasis	25.04 (1.57–400.82)	0.023	Eliminated by Forward:LR	
Bosniak classification Diameter of tumor	120.08 (0.00–905.05) 4.97 (0.90–27.29)	0.403 0.065	/ Eliminated by Forward:LR	

## Discussion

In this study, we found that unilocular cRCC has a lower pathological stage and Fuhrman grade and a better prognosis than purely solid RCC.

The cystic component in RCC has been the focus of scholars for a long time. Hartman first reported unilocular cRCC and divided cRCC into 4 categories: (1) intrinsic multiloculated growth; (2) intrinsic unilocular growth; (3) cystic necrosis; (4) origin from the epithelial lining of a preexisting simple cyst^1^.

However, this classification has not been widely accepted by pathologists. Cystic necrosis, for example, is actually caused by the imbalance of tumor growth and insufficient blood supply. It is not only found in various pathological types of RCC, but also common in other malignant tumors. The existence of malignant transformation of simple cysts is still debatable and there are rare related reports. Therefore, many pathologists believe that the above two subtype should be excluded from the subcategories of cRCC, which means that cRCC should only include the unilocular cystic and the multilocular cystic morphologically^14,15^.

With the continuous increase of relevant researches, multilocular cRCC has gradually become the focus. Both the 2004 revised and 2016 WHO guidelines only defined multilocular cRCC without mentioning unilocular cRCC^4,16^. Although unilocular cRCC has been reflected in some studies, there is no specific study with a large sample size. Meanwhile, many studies did not emphasize the classification of cRCC subtype. As a result, the two concepts of continuously redefined “MCRNLMP” and “cystic renal cell carcinoma” have been implemented without coming into conflict, which has caused a lot of confusion for researchers and clinicians.

In some studies, it was found that there were many papillary RCC in unilocular cRCC^1,17^. This is consistent with our findings. Group A had significantly more papillary RCC than group B, which is somewhat inconsistent with the findings of previous studies in that, the main histology of unilocular cRCC in our study is clear cell carcinoma, not papillary RCC. This discrepancy may be due to the small number of samples used in previous studies^1,17^. At the same time, Hartman also noticed that a few cysts discovered on CT imaging are actually extensive necrosis and tumor necrosis is one of the negative indicators in many prognostic models of RCC^18^. In this study, we defined unilocular cRCC, which excludes necrotic cysts through surgical pathology. However, it is worth noting that it is not reliable to determine whether the cystic component is a true cyst or a necrotic cyst only though preoperative CT imaging. For instance, radiologists with more than 3 years of work experience reviewed the imaging data in this study, but among the 83 cases initially judged as true cysts, 9 cases (11%) were still pathologically confirmed as necrotic cysts. Some studies have shown that MRI may be more advantageous than CT in the differential diagnosis of true cysts^19^ but due to lack of relevant imaging data, we did not investigate that.

Numerous studies on MCRNLMP have shown that the malignancy of this type of tumor is low, and no recurrence or metastasis have been reported so far^2,3^. Therefore, its definition is changed from “cancer” to “neoplasm”. However, of the 74 cases of unilocular cRCC in our study, 1 patient died of metastasis of the RCC. Although no distant metastasis was found at the time of diagnosis, 3 cases of lymphatic metastasis were found in surgical pathology of lymphadenectomy. It can be seen that although the unilocular cRCC is less malignant than the purely solid RCC, its prognosis is not as excellent as that of the MCRNLMP defined by the WHO in 2016.

In this study, we did not set the cut off of the cystic component for unilocular cRCC like other studies but included all RCC with true cystic components. But we found that the cystic proportion of 1 case who dead of RCC in group A was not low (52%), and the cystic proportion of 1 case who had lymphatic metastasis at surgery in group A also reached 78%. We believe that unilocular cRCC even with a high cystic proportion also probably had lymphatic metastasis at the time of diagnosis and recurrence after surgery. Although the presence of cystic components in RCC might predict lower malignant potential, the correlation between high cystic proportion and the mostly indolent outcome has not been fully proven, studies with larger sample sizes are needed in the future.

In previous studies conducted before and after 2016, MCRNLMP was not clearly distinguished from other types of cystic RCC^6–10^. Given that the MCRNLMP is a rare type and was differently defined in previous studies, the true incidence of MCRNLMP was uncertain. According to some recent studies, it can be inferred that it accounts for roughly 1% of RCC^4,5^, while cRCC account for 15%^1,20,21^. Defining the MCRNLMP as being similar with all other types of cRCC and making survival comparison with solid RCC certainly will mask the relatively poor prognosis and relatively high malignancy of other types of cRCC such as the unilocular cRCC. As a result, when making clinical decisions, surgeons may underestimate the malignancy of cRCC other than MCRNLMP. It is a bot puzzling that although the pathological stage and Fuhrman grade of 74 unilocular cRCC cases are relatively low, 3 cases (4.1%) still have lymphatic metastasis at surgery and the difference with that of solid RCC (6.2%) is not significant (*p* = 0.501). We speculated that this may be due to the relatively small sample size. It may also be because that 3 cases of tumors were large in size. The average maximum diameter of the tumors in group A was 4.45 ± 1.10 cm, while that of 3 cases with lymphatic metastasis were 6.57 cm, 6.37 cm, and 6.63 cm, respectively.

The excellent prognosis of MCRNLMP may suggest that the existence of the cystic component of renal carcinoma is somehow related to a favorable prognosis. According to existing researches, it can be seen that the prognosis of MCRNLMP, unilocular cRCC, and solid RCC shows a decreasing trend^2,11,12^. Therefore, the clinical treatment and follow-up decisions of them should be made pertinently. Many studies have pointed out that for MCRNLMP, nephron sparing method should be used as much as possible^22,23^. In view of the findings of this study, we recommend that unilocular cRCC should also be treated with nephron sparing approaches when feasible. Radical nephrectomy is still needed when the tumor is large, and regional lymph nodes should be removed if the preoperative imaging shows the sign of enlarged lymph nodes. However, our proposal and specific implementation plan need to be confirmed by further prospective studies.

For most cases, the diagnosis of different subtype of cRCC based on imaging data ultimately needs to be confirmed by postoperative pathology, which means that clinical decisions on nephron sparing or radical approaches have always been made. Clarifying that the tumor belongs to a specific subtype of cRCC can only be used as a guide for postoperative follow-up or retrieval reoperation. Therefore, it is particular important to judge different subtype by preoperative imaging, especially to identify MCRNLMP and necrotic cysts. Several relevant studies published may help on this issue, but there is still room for improvement in the accuracy of diagnosis^24^.

There are several limitations to our study. (1). This study is retrospective in nature; the pathological features were summarized based on the surgical pathology reports. We were unable to personally obtain tumor specimens for further analysis, such as cyst fluid properties, more comprehensive immunohistochemistry, etc. (2). As a result of the intention to investigate unilocular cRCCs and the total number and the number of cancer-specific deaths in group A were small, the optimistic cut off of cyst/solid ratio related to prognosis could not be calculated. (3). Twenty patients were excluded due to incomplete imaging data, which may have caused selection bias to the outcomes. (4). In order to eliminate confounders, we performed PSM in 761 solid RCC cases, and eventually enrolled 144 cases in the control group. Therefore, when performing Cox regression, the Goodness of Fit may be not ideal enough. Although the PSM method adopted to select matching objects is random, the solid RCC group still has the possibility of underrepresentation. However, the characteristics of solid RCC in other similar studies with larger sample size can be corroborated^2,7,8^.

## Conclusion

We reported the first large series of long-term survival of unilocular cRCC. Compared with purely solid RCC, unilocular cRCC have a lower Fuhrman grade and pathological stage and a better prognosis but some patients still probably have lymphatic metastasis at the time of diagnosis and may have postoperative relapse, which is different from that in MCRNLMP. The presence of unilocular cyst should be documented in the pathology report like the multilocular one, and when making clinical decisions and follow-up plans for cRCC, the different subtype of cRCC could be taken into consideration as a factor.

## Human and/or animals participants

This study is a review of existing data and does not involve any studies with human participants or animals performed by other authors.
